# Multifocal pleural capillary hemangioma: a rare cause of hemorrhagic pleural effusion-case report

**DOI:** 10.1186/s12890-021-01507-5

**Published:** 2021-05-10

**Authors:** Naifu Nie, Zhulin Liu, Jun Kang, Li Li, Guoqiang Cao

**Affiliations:** Department of Respiratory Disease, Daping Hospital, Army Medical University, Chongqing, 400042 China

**Keywords:** Multifocal pleural, Capillary hemangioma, Hemorrhagic pleural effusion, Azathioprine

## Abstract

**Background:**

Capillary hemangioma can be found in many organs, but rarely in pleura. Previously, only localized pleural capillary hemangioma cases have been reported. Corticosteroids are the most commonly recommended drugs in capillary hemangioma.

**Case presentation:**

Here, we present a case of a young woman with recurrent hemorrhagic pleural effusion. Despite repeatedly thoracentesis, the routine examinations, including chest computed tomography (CT) scan, pleural effusion biochemical test, and cytology all failed to make a definite diagnosis. Thus, single port video-assisted thoracoscopy (VATS) was then performed. Numerous nodules arising from the parietal pleura were found, and biopsies showed multifocal pleural capillary. However, recurrent pleural effusion was successfully managed by oral azathioprine, after failure of dexamethasone treatment.

**Conclusions:**

To our knowledge, this is the first case of a patient with recurrent hemorrhagic pleural effusion masquerading as malignant pleurisy, but in fact caused by multifocal pleural capillary hemangioma.

## Background

Capillary hemangioma is a neoplasm of vascular proliferation, which is benign but may be multifocal. Capillary hemangioma is commonly found in facial skin, subcutaneous tissue and oral mucosa, although it can be found in any organs [1, 2]. In the current study, we described a case of multifocal capillary hemangioma in a young female with recurrent hemorrhagic pleural effusion and undertake a literature review of such unusual tumors. As far as we know, this is the first report of hemorrhagic pleural effusion caused by multifocal pleural capillary hemangioma.

## Case presentation

**Fig. 1:**
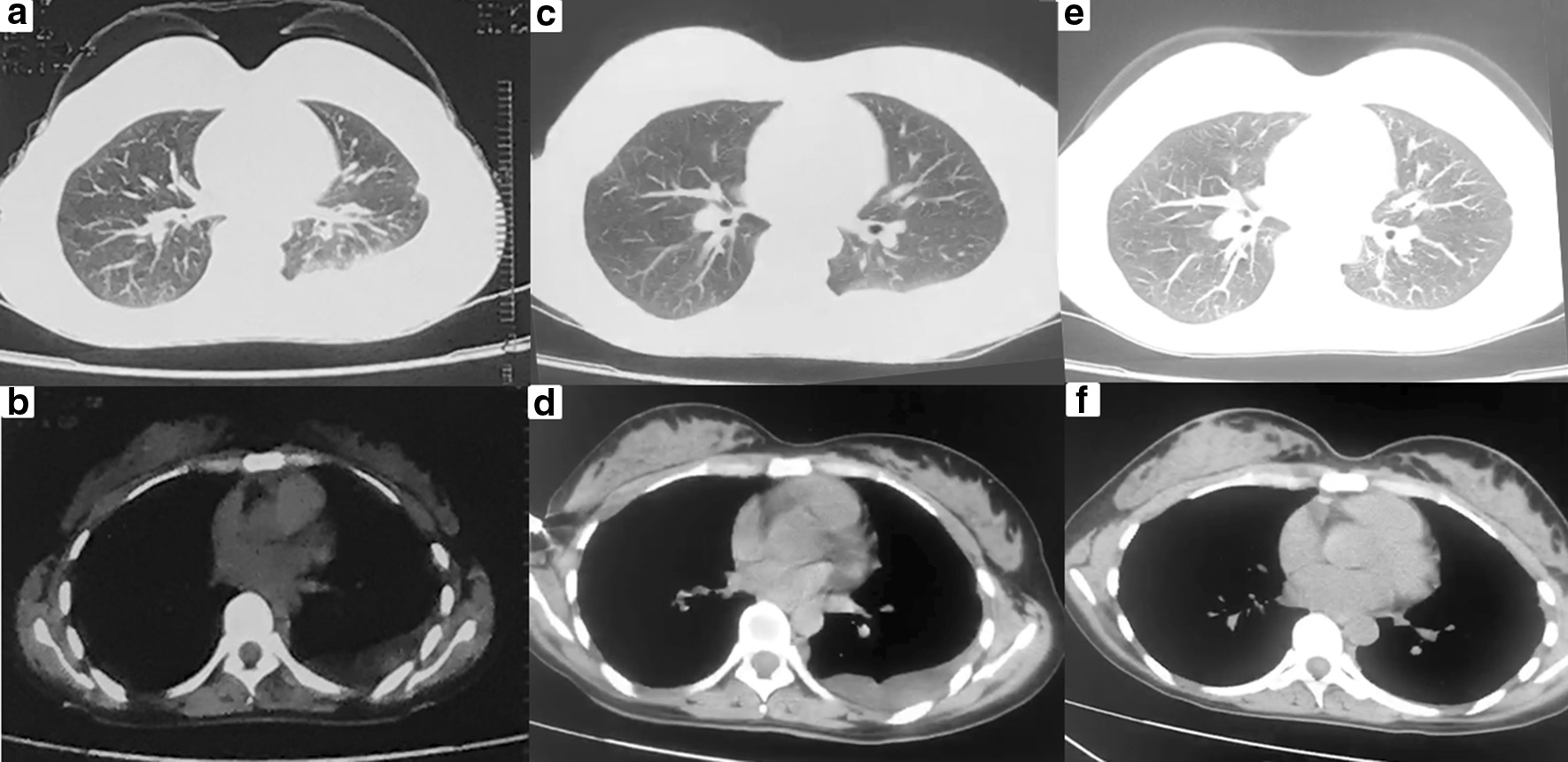
**a**, **b** CT scans revealed that left pleural effusion without any nodule in the lung fields. **c**, **d** The pleural effusion did not absorb well after 4 weeks of oral corticosteroids. **e**, **f** CT scan revealed no pleural effusion after 12 months of oral azathioprine

**Fig. 2:**
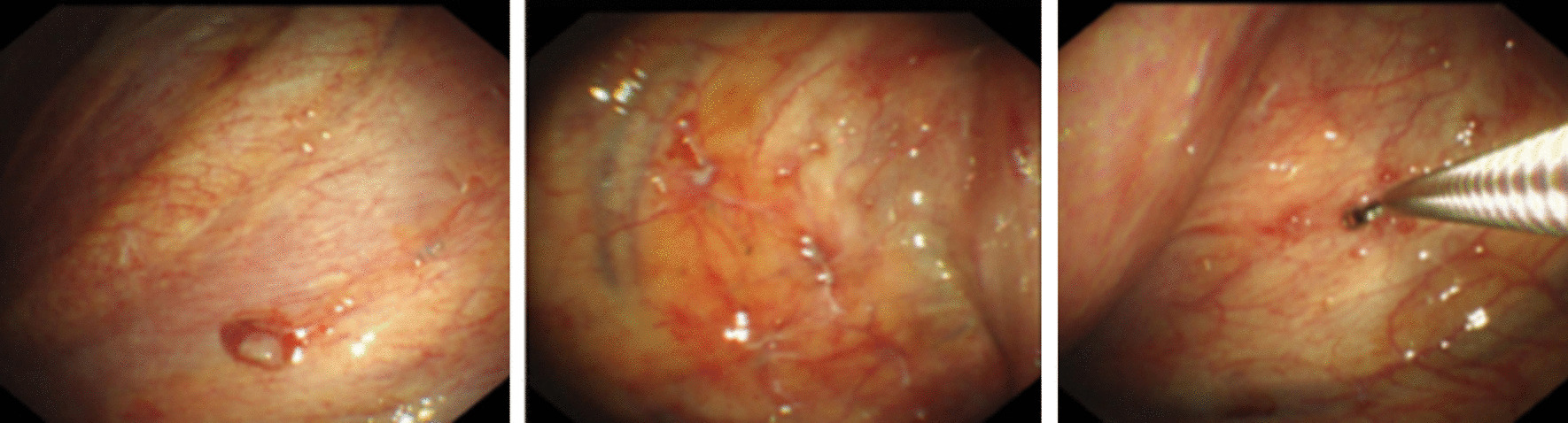
Single port video-assisted thoracoscopy (VATS) showing a large number of nodules on parietal pleura, and biopsy was performed on multiple sites

**Fig. 3:**
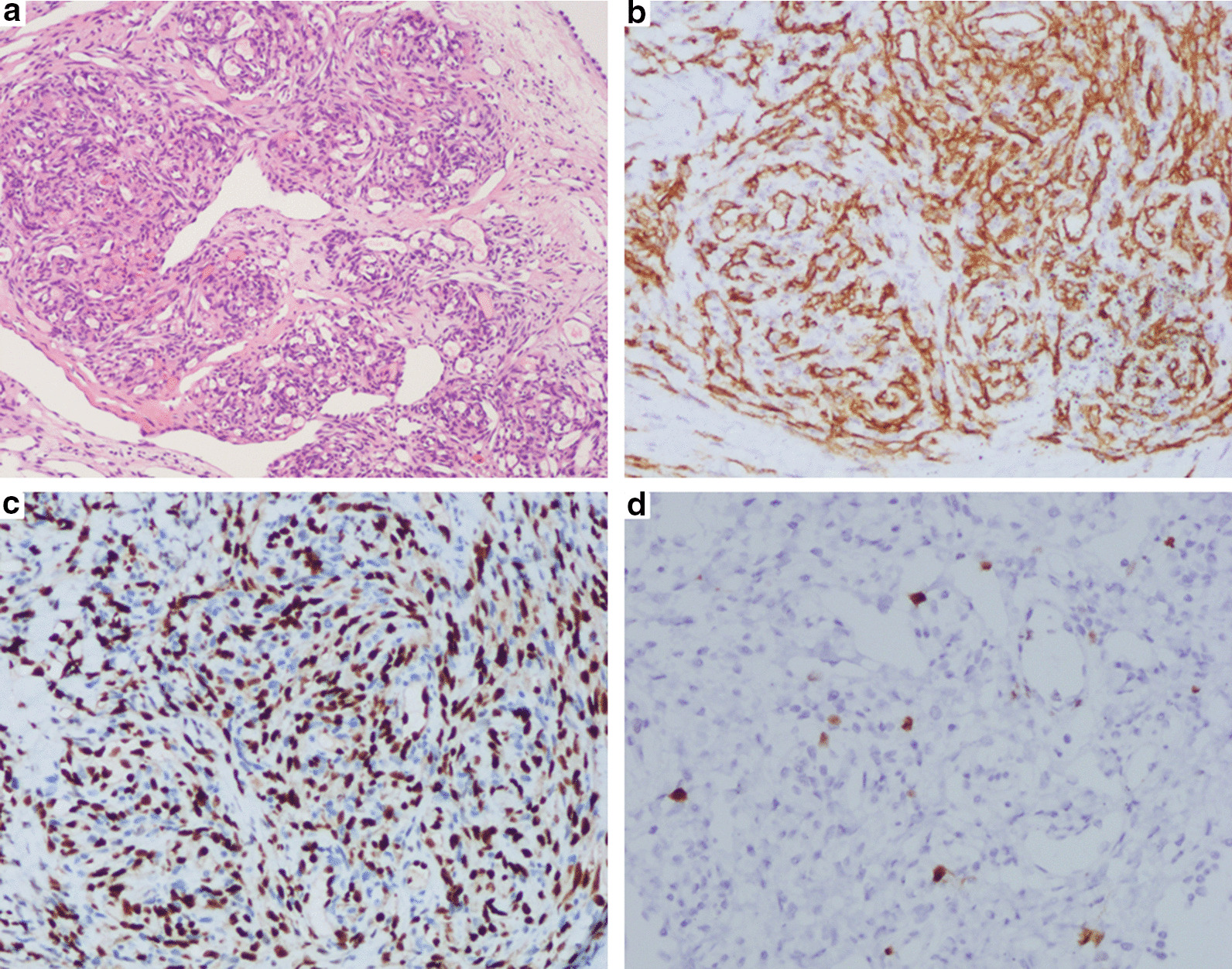
**a** Microscopically, the tumor was composed of capillary-sized vessels filled with red blood cells, and the lining endothelial cells showed no significant atypia (hematoxylin–eosin staining; × 100). **b**–**d** Immunohistochemical analysis on CD31 and ERG, and Ki-67, respectively

We report the case of a 32-year-old non-smoker female, who presented with mild dyspnea for two weeks. She denied cough, hemoptysis, fever and weight loss. Serologic tumor markers, including carcinoembryonic antigen (CEA) and α-fetoprotein (AFP), did not reveal any abnormalities. Tuberculin purified protein derivative (PPD) test of the patient was negative. A computed tomography scan (CT) of the chest revealed left pleural effusion, and there was no evidence of mediastinal lymphadenopathy and the lung fields were clear (Fig. 1a, b). Although undergoing repeatedly thoracentesis, the hemorrhagic pleural effusion was recurrent refilling. The routine biochemical test of pleural effusion showed that it was exudative, while no malignancy was detected on cytology examination. Thus, single port video-assisted thoracoscopy (VATS) was then performed. Thoracoscope showed a large number of nodules arising from the parietal pleura, with a tenacious texture and a clear margin. Biopsy was performed on multiple nodules, with a little bleeding (Fig. 2). Microscopic examination revealed the nodules comprised a conglomerate of capillary vessels, and some of the vascular spaces lined by proliferating endothelial cells. No significant atypia was found in the lining endothelial cells (Fig. 3a). Immunohistochemical (IHC) analysis showed CD31 and erythroblast transformation specific regulated gene-1(ERG) positive, and Ki-67 proliferation index was 2% (Fig. 3b–d). The biopsy was suggestive of capillary hemangioma. Dexamethasone (10 mg/day)was injected into the thoracic cavity for three consecutive days, and then the patient began to receive prednisone acetate (30 mg/day). Unfortunately, both topical and systemic dexamethasone were failure to control pleural effusion. The chest CT scan showed that medium-volume still exist after 4 weeks of oral corticosteroids (Fig. 1c, d). Hence, the patient stopped corticosteroids and switched to oral azathioprine 100 mg ever day. No side effect associated with azathioprine occurred. The patient was followed up regularly and the pleural effusion was gradually reduced. At the 12 months of follow up, reexamination with CT scan revealed no pleural effusion (Fig. 1e, f).

## Discussion and conclusions

Capillary hemangioma is an even rare congenital disease, which is thought to be associated with the imbalance between proangiogenic factors and angiogenesis inhibitors [3, 4]. Pleural capillary hemangioma was extremely rare, and all of the reported cases previously are the localized lesion [5]. As far as we know, multifocal pleural capillary hemangioma has not been reported in the literature.

Multifocal pleural capillary hemangioma is more like a localized capillary hemangioma with multiple lesions, which was different from the diffuse neonatal hemangiomatosis [6]. Most previous reports describe pleural hemangiomas as being asymptomatic, and a few presenting with cough or short of breath. They are usually diagnosed incidentally or when spontaneous rupture causing hemorrhage or hemorrhagic pleural effusion [7]. As might be expected, the patient in our case presented with recurrent accumulation of one-sided pleural effusion masquerading as malignant pleurisy. Although the precise cause of pleural effusion remains unclear, the capillary tumor vessels might play a causative role in increasing microvascular permeability or pleural inflammatory processes [8].

Diagnosis of hemangioma mainly depends on imaging and pathological examinations. Multifocal capillary hemangiomas may present numerous nodules on the pleural surface with pleural thickening on CT scan [9]. However, chest CT doesn’t always reveal findings suggestive of hemangiomas, especially those characterized by small pleural nodules. Medical thoracoscopy is an important method for the diagnosis of pleural hemangioma. Histopathological examination and IHC are essential. Microscopically, capillary hemangiomas consisted of vascular spaces lined by proliferating endothelial cells, with few dilated channels. The sparse inflammatory cells and fibrocollagenous stroma were found in the vascular spaces. The neoplasm showed no cellular atypia or mitosis [3, 10]. In our case, numerous nodules of pleura can found through thoracoscopy, which were biopsied and diagnosed as pleural capillary hemangioma.

The majority of hemangiomas are harmless cutaneous lesions, but approximately 10% of hemangiomas need a therapeutic approach because of their location, size, or behavior [11]. The treatments were mainly based on the location and quantity of the lesions [12].Besides, neoplasm size and adjacent structures, the age should also be considered [13]. For localized hemangioma, surgical resection is the most common approach. While for multifocal pleural capillary hemangioma, individualized and multimodal treatment approach may be necessary, including dry ice cryotherapy, sclerosing agent injection, radiotherapy, vascular embolism, surgical excision and drugs therapy [14, 15]. Drugs used include corticosteroids, interferon-alfa, propranolol and immunosuppressive agents [6, 15].

Steroid hormones are most commonly used in the treatment of hemangioma, which may promote regression of the proliferating hemangioma by inhibition of cytokines, regulating of hemangioma proliferation and involution [15]. High doses of systemic or intralesional steroids are the first-line treatment [11]. In vitro studies, dexamethasone may be the best choice for treating hemangioma [14]. Local use of dexamethasone can reduce capillary permeability and relieve exudation. Immunosuppressive agents can be used in cases which did not respond to corticosteroids [16]. In our case, dexamethasone was not effective. However, after oral application of azathioprine, pleural effusion was reduced gradually and completely disappeared at the 12 months of follow up. Hence, azathioprine may be one of choice for multiple pleural hemangiomas.

To conclude, capillary hemangioma in the pleura is extremely rare, for recurrent hemorrhagic pleural effusion, the possibility of benign tumor such as hemangioma should be considered. Thoracoscopy is an important method for diagnosis. Due to the rarity of multifocal plural capillary hemangioma, the treatment still needs to be further explored. In addition to corticosteroids, immunosuppressive agents may also be potential therapeutic drugs. Hence, reporting of our case and sharing the treatment experiences may be helpful to improve for clinical therapeutic strategy.

## Data Availability

Not applicable.

## Abbreviations

CT: Computed tomography
VATS: Video-assisted thoracoscopy
PPD: Purified protein derivative
CEA: Carcinoembryonic antigen
AFP: α-Fetoprotein
IHC: Immune- histochemical
ERG: Erythroblast transformation specific regulated gene-1
